# Association Between Diet, Sociodemographic Factors, and Body Composition in Students of a Public University in Ecuador

**DOI:** 10.3390/ijerph22071140

**Published:** 2025-07-18

**Authors:** Angélica María Solís Manzano, María Victoria Padilla Samaniego, Verónica Patricia Sandoval Tamayo, Edgar Rolando Morales Caluña, Katherine Denisse Suarez Gonzalez, Tannia Valeria Carpio-Arias, Patricio Ramos-Padilla

**Affiliations:** 1 Nutrition, Dietetics, Biotechnology and Food Analysis Research Group, Milagro State University, Milagro 091701, Ecuador; mpadillas@unemi.edu.ec (M.V.P.S.); vsandovalt@unemi.edu.ec (V.P.S.T.); ksuarezg@unemi.edu.ec (K.D.S.G.); 2 Faculty of Social Services, Nutrition and Dietetics Program, Universidad Estatal de Milagro, Milagro 091050, Ecuador; emoralesc4@unemi.edu.ec; 3 Research Group on Food and Human Nutrition GIANH, Polytechnic School of Chimborazo, Riobamba 060104, Ecuador; patricio.ramos@espoch.edu.ec

**Keywords:** body composition, sociodemographic factors, diet, university students, Public health

## Abstract

Body composition is associated with multiple factors. The main objective of this study is to determine the association between diet and sociodemographic factors on the body structure and composition of university students at a public university in Ecuador. This cross-sectional study allowed for the collection of detailed body composition and dietary data from 204 students (41.7% men and 58.3% women, with an average age of 23.3 ± 4.4 years). The study was conducted using validated questionnaires and bioimpedance techniques. Statistical analysis included ANOVA tests, complemented by a PCA-Biplot, to examine the relationships between study variables. Statistical analysis revealed that men’s birthplace had a significant impact on several body measurements, such as hip circumference and weight, but no significant differences were observed in body structure and composition based on nutrient intake. Furthermore, larger upper-arm circumference in women was correlated with higher fat intake. The results of the multivariate analysis indicated a differential influence of dietary components on body composition. The study highlights the need for nutritional intervention strategies and educational programs that consider the diversity of students’ backgrounds to promote healthy habits and mitigate the negative effects of eating habits and irregular physical activity patterns on their health and body composition.

## 1. Introduction

Diet, a central pillar of health, can be influenced by several factors such as educational level and socioeconomic context. However, in certain population groups such as university students, it may also be affected by other elements, including lack of time, physical activity, and limited financial resources [1,2]. The diet of university students is not merely a matter of personal choice or food availability. It is also deeply influenced by factors such as stress, which is inherent to academic responsibilities and can lead to noticeable changes in dietary patterns, thereby affecting physical well-being [3,4]. These changes can also promote a sedentary lifestyle [5] and harmful eating behaviors, increasing the risk of conditions such as hypertension and obesity [6].

Studies have shown a significant relationship between the consumption of high-calorie-density foods and weight gain among students, which also affects factors of body composition [7]. This underscores the importance of dietary quality in cardiovascular health [8,9]. Body composition is the result of a complex network of factors, including purely biological ones such as various genetic mechanisms that cause imbalance [10] as well as modifiable factors like physical activity [11] and diet, encompassing meal timing and circadian rhythm disruptions [12].

Previous studies have demonstrated that a higher educational level among university students may be associated with healthier eating patterns, reflecting better dietary quality and, consequently, more optimal body composition [13]. Additionally, differences have been observed based on socioeconomic factors, with male students tending to exhibit poorer habits compared to female students [14]. Nutritional status differences and weight changes among health sciences students not only reflect individual eating habits but also result from a complex interplay with socioeconomic and cultural factors [15]. Cheli Vettori et al. [16] highlight how these factors influence dietary choices and, consequently, students’ cardiovascular health and overall well-being. This dynamic is exacerbated by inequalities in access to healthy foods and opportunities for physical activity, both of which are closely tied to students’ socioeconomic status. Furthermore, research suggests that academic stress may intensify these effects, leading to sedentary behaviors and poor dietary choices that jeopardize cardiovascular health [5].

Therefore, it would be thought that in adult university students, specific sociodemographic conditions and the resulting changes in diet and body composition are associated with an increase in metabolic risk factors [17].

Therefore, it is important to conduct this study to contribute to improving knowledge about these associations, especially by determining the differences between groups of university students based on sociodemographic characteristics. Studies of this type are scarce in the Ecuadorian population, and their importance relates to planning interventions tailored to the living conditions of university students.

The main objective of this study is to determine the association between diet and sociodemographic factors on the body structure and composition of university students at a public university in Ecuador.

## 2. Materials and Methods

This was a non-experimental, cross-sectional study. Anthropometric data were collected, and bioelectrical impedance techniques were used to assess body composition. Information was gathered through questionnaires designed to capture sociodemographic data such as age, sex, ethnicity, marital status, place of birth, residence, and parental origin, all of which could influence students’ dietary patterns and their body structure and composition. Detailed dietary data were obtained using the validated block screening questionnaire [18].

The sample for this study consisted of 204 students from the Universidad Estatal de Milagro during the 2022–2023 academic year. The study employed a non-probabilistic convenience sampling method, aiming to represent a diversity of perspectives.

Probabilistic stratified sampling was calculated from 16,000 students with an expected frequency of 50%, a margin of error of 7%, and a confidence level of 95%, obtaining a total of 194 participants. A 10% increase was added for possible losses, resulting in 204 students. The calculator version of the Epi Info program, version 7.2.6, was used for the calculation.

The selection criteria for the study’s students were based on an open call for participation, issued by the university. Once interested parties contacted the study team, their eligibility was verified, informed consent forms were shared, and if acceptance was confirmed, the student signed the application. This process continued for three months until the calculated sample was reached.

### 2.1. Body Composition and Structure

Body composition and structure were assessed using anthropometric evaluations that included measurements of body weight, skinfold thickness at four sites (triceps, subscapular, biceps, and suprailiac), body circumferences at three locations (relaxed arm, waist, and hip), and bone diameters at three points (transverse thorax, anteroposterior thorax, and biacromial diameter). In addition, bioelectrical impedance analysis was used to measure the total body fat percentage and total muscle mass percentage. All measurements were conducted using standardized techniques according to the ISAK protocol [19].

The bioimpedance analyzer and software used in this study were the InBody 120 (InBody South Africa, Pretoria, South Africa) and the Lookin’Body 120 software. The InBody 120 is a multi-frequency segmental electrical bioimpedance (DSM-BIA) device of Korean origin. The reliability and validity of the InBody device have been evaluated in various scientific studies [20]. Although most research has focused on similar models such as the InBody 230, the InBody 120, which shares similar technology, would also offer comparable reliability.

The reported reliability values for the InBody 230 are as follows: intraclass correlation coefficients (ICCs): ≥0.98 for body fat percentage (BF%), fat mass (FM), and fat-free mass (FFM). The validity of the InBody devices has been demonstrated through significant correlations: r = 0.94–0.99 between the InBody 230 and DEXA for fat mass, BF%, and total FFM.

### 2.2. Dietary Characteristics

Diet was evaluated using the block screening questionnaire [21], which assessed intake of fats, fruits, vegetables, and fiber (see Annex A). According to this screening tool, scores were established for fat consumption (“fat points”) and for the consumption of fruits, vegetables, and fiber (“fruit and fiber points”).

The block screening questionnaire was initially published in 1986, and a reduced version was published in 2000; this version was used in this study [18].

The block screening questionnaire has demonstrated excellent reliability in studies with Spanish-speaking populations, with coefficients ranging from 64% to 94%, depending on the component assessed [22].

### 2.3. Univariate Statistical Analysis

Statistical analyses were conducted to identify significant correlations and trends between diet, sociodemographic factors, and the students’ body structure and composition. For data visualization, boxplots were used, which clearly illustrated dispersion, symmetry, and the presence of outliers across the different body composition variables as grouped by dietary and sociodemographic factors. This visualization allowed for preliminary group comparisons prior to applying statistical tests.

Subsequently, one-way ANOVA tests were conducted to identify statistically significant differences in means across the groups categorized by sex, dietary scores (fat and fruit/fiber scores), and sociodemographic variables (birthplace, place of residence, and parental origin) [23]. Whenever statistically significant differences were observed (*p* < 0.09), a Bonferroni post hoc test was applied to identify which pairs of groups exhibited these differences, thereby ensuring control over Type I error in multiple comparisons.

The proposed methodology was also inspired by studies conducted by Hendricks et al. [4,24], who applied similar approaches in analyzing specific populations, adapting data collection and analysis tools to address their particular research questions.

### 2.4. Multivariate Analysis

A biplot is a technique that graphically represents a dataset in two-dimensional space. In this representation, individuals appear as points and variables as vectors [25]. One of the main advantages of biplots is their ability to reveal relationships between variables: acute angles between vectors suggest a direct correlation, obtuse angles indicate an inverse association, and 90-degree angles imply independence between variables [26].

The objective of this section was an exhaustive analysis of the cohort without gender distinctions, exploring the relationships between study variables through a principal component analysis biplot (PCA-Biplot) [27]. This aimed to determine whether diets evaluated based on scores in fats, fruits, vegetables, and fiber, and sociodemographic factors contribute to variations in students’ body structure and composition. To achieve this, angles between vectors were analyzed to identify positive correlations in acute angles and negative correlations in obtuse angles, in accordance with multivariate analysis principles.

This methodological approach enables an understanding of how various aspects of daily life, including eating habits and sociodemographic conditions, interact with individual body morphology, which is especially relevant in developmental contexts such as Ecuador. The interpretation of angles between vectors will allow for the identification of association patterns among variables, offering valuable insights for future advances in understanding human biology and its interactions with demographic factors.

### 2.5. Ethical Considerations

This study was approved by the Human Research Ethics Committee of the Escuela Superior Politécnica de Chimborazo, under the code IO-07-CEISH-ESPOCH-2023.

## 3. Results

The sample was composed of 41.67% men and 58.33% women, with a mean age of 23.34 years, and a range of 19 to 55 years. The median age was 22 years, the mode was 21 years, and the standard deviation was 4.38 years.

Table 1 presents a description of the anthropometric characteristics of the study group, which shows a mean skinfold thickness of 80 mm, a bone diameter of 102.47, an upper arm ratio of 32.37 cm, a waist circumference of 84.17 cm, a hip circumference of 108.13 cm, a fat mass of 39.93%, a muscle mass of 23.22%, and a weight of 72.82 kg.

Regarding dietary patterns, 22% of the students were observed to have a very high fat intake, followed by 7% with a high-fat diet. Furthermore, 16% of the population maintained a medium-fat diet, while 26% followed a low-fat diet. On the other hand, 29% of the participants reported following an almost completely fat-free diet.

Regarding fruit, vegetable, and fiber consumption, only 2% of the respondents met the standards for a nutrient-dense diet. In contrast, more than 19% of the students maintained a diet that required greater supplementation with vegetables and grains. Furthermore, more than 78% of the respondents were found to follow a nutrient-reduced diet.

Regarding dietary assessment based on fat content, a correlation with arm circumference was observed in women. Those who followed a low-fat diet showed significantly larger arm circumferences compared to those who consumed a medium-fat diet (Table 2).

**Figure 1:**
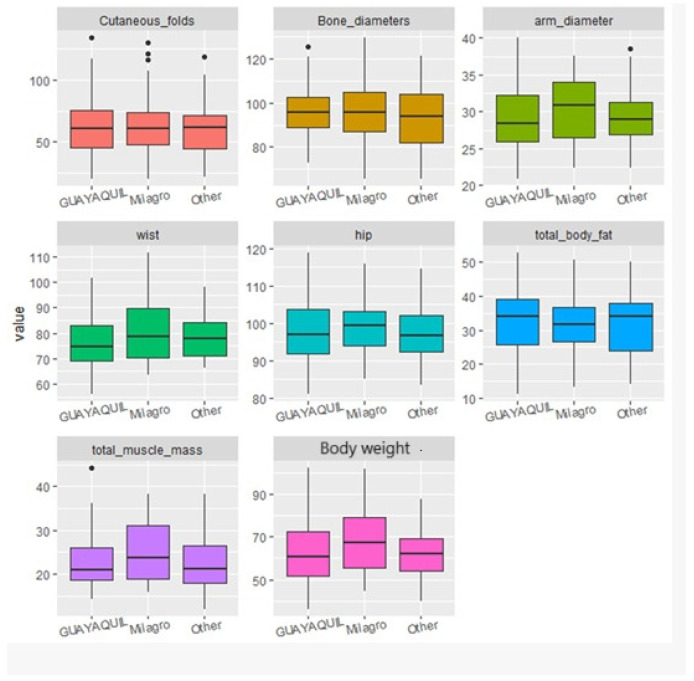
Boxplots of body structure and composition according to the respondent’s place of birth.

**Figure 2:**
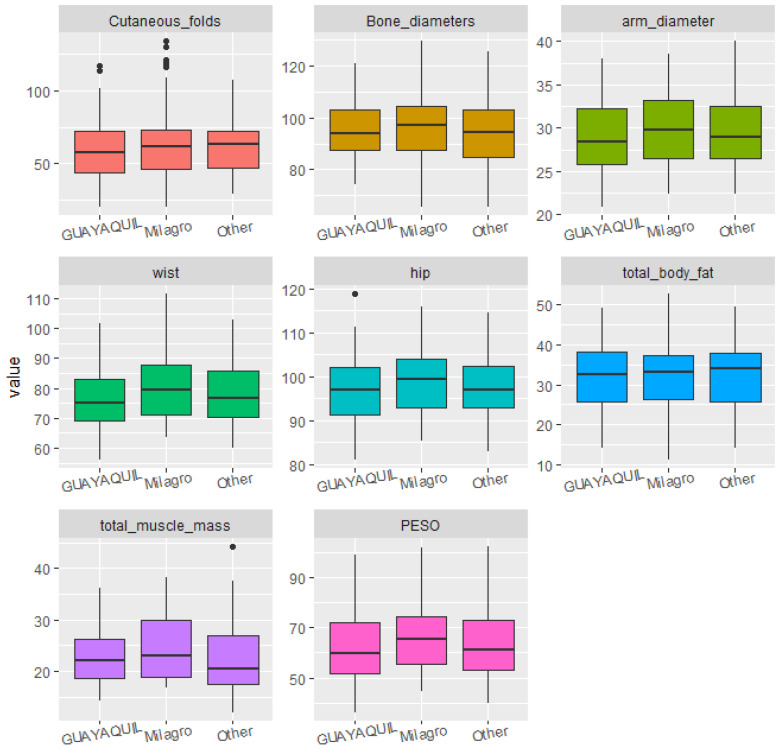
Boxplots of body structure and composition according to the respondent’s place of residence.

The students’ place of birth and place of residence did not significantly impact the participants’ body measurements (Figure 1 and Figure 2).

**Figure 3:**
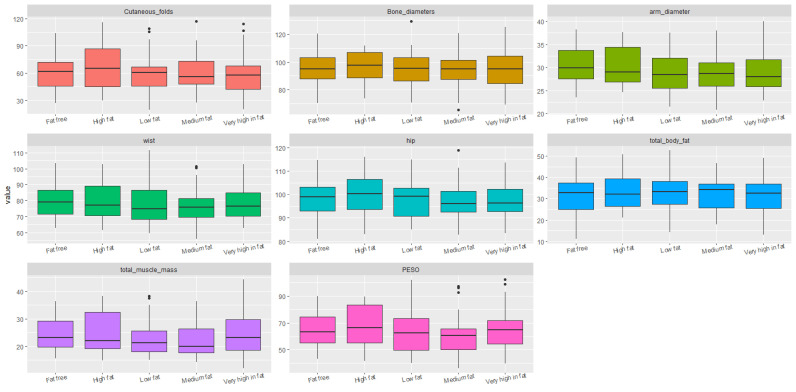
Boxplots of body structure and composition according to dietary scores for high-fat foods.

**Figure 4:**
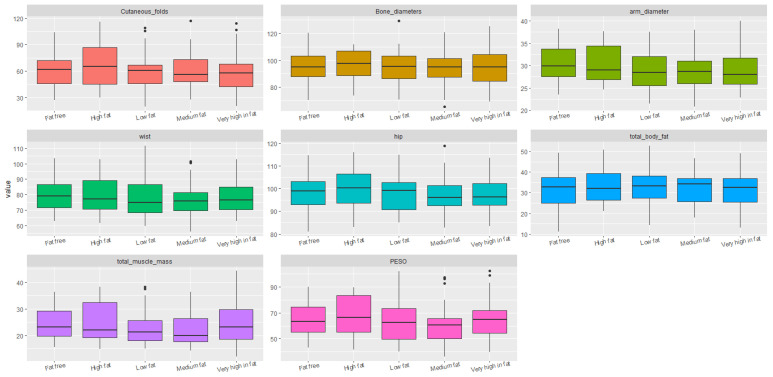
Boxplots of body structure and composition according to dietary scores for fruits, vegetables, and fiber.

No significant differences were found between students in relation to their intake of fat, fruits, vegetables, or fiber (Figure 3 and Figure 4).

However, among male students, significant differences were observed in the sum of the four skinfolds, hip circumference, and total body fat percentage (Table 3).

**Figure 5:**
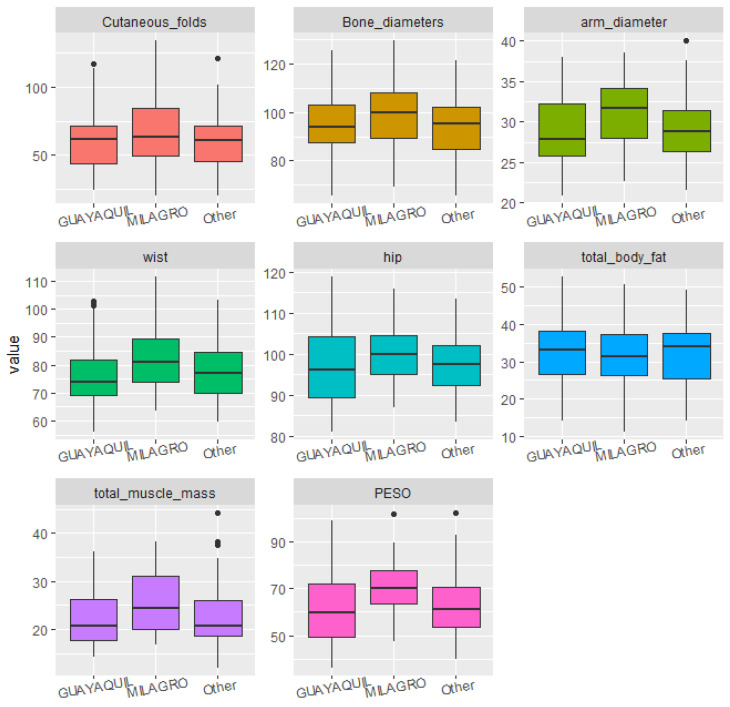
Boxplots of body structure and composition according to the father’s place of origin.

**Figure 6:**
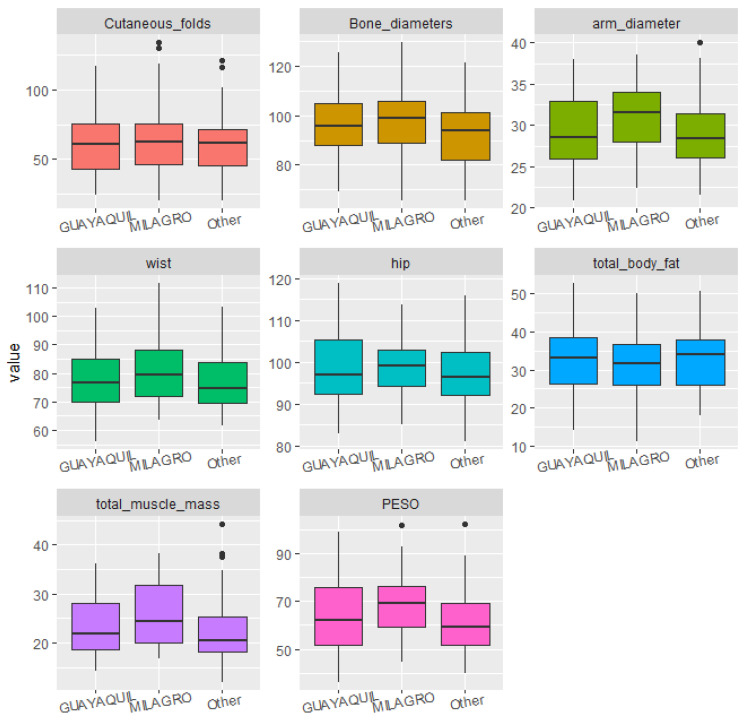
Boxplots of body structure and composition according to the mother’s place of origin.

The father’s place of birth did have a significant impact on total body mass percentage in men (Figure 5). Those whose fathers were originally from Milagro had a significantly higher body mass percentage than those whose fathers were from Guayaquil (Figure 5). On the other hand, the students’ place of residence, regardless of gender and their mothers’ place of origin, did not show significant differences in body structure, body fat percentage, or weight (Figure 6). The results of the statistical analysis are presented in Table 2, Table 3 and Table 4.

Men born in Milagro had a significantly larger hip circumference compared to those born in other locations, excluding Guayaquil, and also had a higher body weight. Furthermore, men born in Guayaquil and Milagro showed a significantly higher skinfold sum compared to those born in other areas, along with a higher percentage of total body fat.

**Figure 7:**
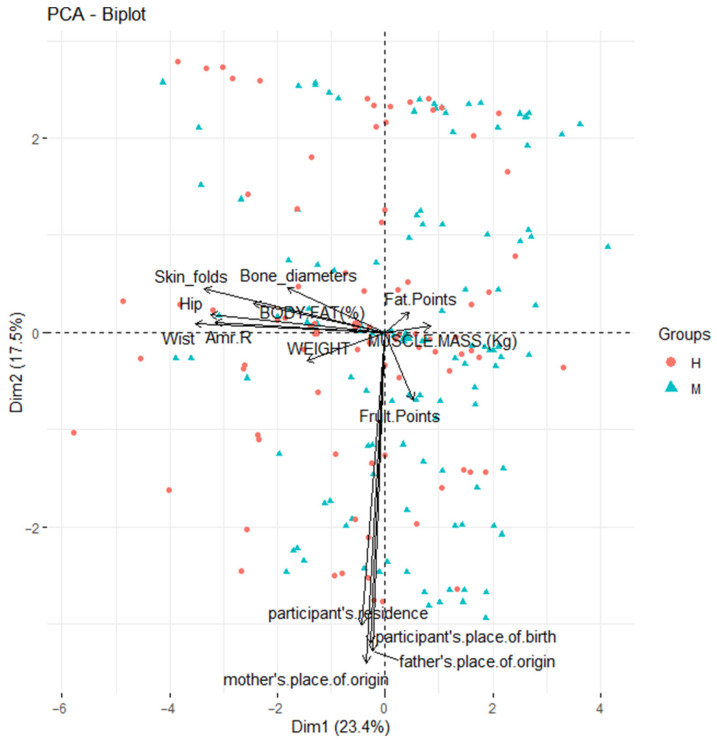
*PCA-Biplot:* Correlation of the variables studied and changes in body structure and composition plane 1–2.

**Figure 8:**
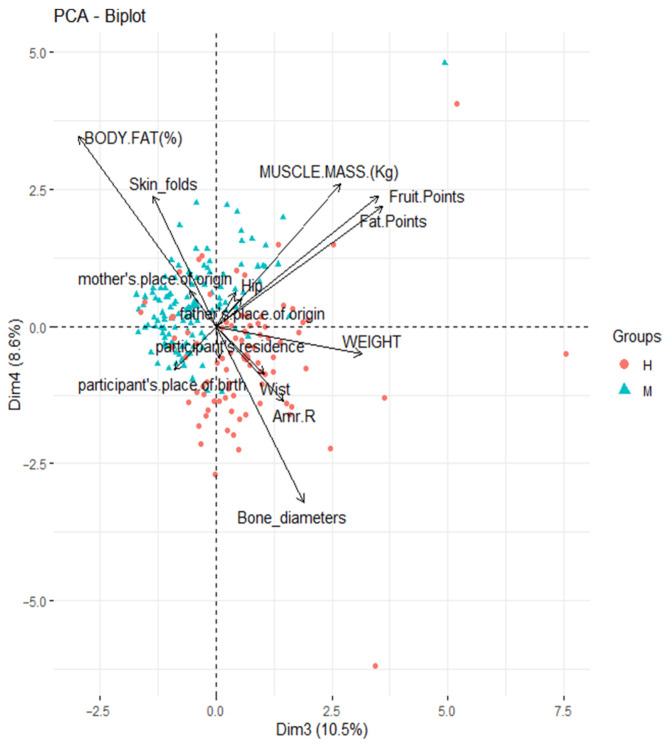
*PCA-Biplot:* Correlation of the variables studied and changes in body structure and composition plane 3–4.

As shown in Figure 7 and Figure 8, the accumulated inertia reaches approximately 60% in the four principal components, considering the 14 variables analyzed in this study.

According to the data presented in Table 5, it is observed that sociodemographic variables show greater association with the first component, while the sum of the four skinfolds, arm, waist, and hip measurements display significant representation in the second component. However, upon examining Figure 7, it becomes evident that the points reflecting the intake of fats, fruits, vegetables, and fiber in the diet are not adequately distributed in the plane defined by components 1 and 2. Furthermore, our analyses revealed that the sociodemographic factors of the participants exhibit a weak or nearly nonexistent correlation with body structure and composition measurements, with the exception of body weight.

The variables related to dietary scores for fats, fruits, vegetables, and fiber were better represented in components 3 and 4. In particular, the muscle mass percentage showed a more robust association with these components. Additionally, the sum of the four bone diameters, percentage of muscle mass, and percentage of body fat were better represented in component 4.

As shown in Figure 8, there appears to be a positive and significant association between the points representing the intake of fats, fruits, vegetables, and fiber, as well as muscle mass percentage. However, the points corresponding to fruit, vegetable, and fiber intake do not appear to be related to total body fat percentage, and the points associated with fat intake do not show a clear association with the sum of the four bone diameters. This suggests a differential influence of dietary components on body composition, where the intake of fats, fruits, vegetables, and fiber may have a positive impact on muscle mass, whereas their relationship with total body fat and bone structure may be less pronounced.

## 4. Discussion

This study aimed to identify the influence of diet and sociodemographic factors on the body structure and composition of university students in public higher-education institutions.

The results showed that only 2% of students reported having a nutrient-rich diet, and 29% followed a fat-free diet. This situation among university students aligns with findings from a study conducted on a student cohort at the University of Valladolid in Spain [28].

In our analysis of food intake and nutritional content, no significant differences were identified between male and female university students. This contrasts with the findings of [29], who conducted a study among medical students at a Mexican public university and found a positive relationship between muscle mass and total energy intake.

Nevertheless, our results revealed an interesting association between a high-fat diet and arm circumference in female students. This correlation suggests that lower fat intake may be linked to greater muscle mass in women, which is consistent with the findings of [8], who identified a significant relationship between the consumption of energy-dense foods and an increase in visceral fat. Additionally, [9] emphasized the connection between overweight, obesity, and irregular levels of physical activity, emphasizing the importance of a balanced diet and an active lifestyle for overall health.

Our findings suggest that both place of birth and socioeconomic context significantly influence body composition in male university students, particularly in measures such as hip circumference and body weight. This influence may be related to regional differences in dietary traditions and the availability of healthy food options. In contrast, a study conducted among 376 children in Jablona found no significant differences in body proportions between urban and rural children [30], indicating that such influences may become more pronounced in older populations, such as university students. This contrast underscores the complexity of the interactions between environment, nutrition, and body composition.

This influence may be attributed to variations in dietary traditions and access to nutritious food across different regions. Furthermore, according to [5], academic stress can alter dietary patterns, leading students to opt for less healthy food choices. These choices can have adverse effects on both physical health and academic performance. This set of findings highlights the complexity of factors that influence the nutrition and health of university students, a central theme in our study.

In relation to cardiovascular health, the results echo the concerns raised by [5,6], in which academic stress may lead to a more sedentary lifestyle and harmful dietary habits, thereby increasing the risk of hypertension and obesity.

Some studies have shown that the geographic origin of family residence and maternal lineage do not significantly influence body proportions or anatomical structure in children and adolescents, as seen in the work of [31]. In our study, we did not observe any effects on body morphology among Ecuadorian university students, regardless of their place of residence or maternal origin, even though Ecuador is a developing country. This finding contrasts with common perceptions about the relationship between socioeconomic determinants and human physical characteristics [32].

This study contributes significant evidence on the influence of diet and sociodemographic factors on university students’ body composition. It underscores the need for the inclusion of nutrition education programs in universities, which could play a pivotal role in improving nutritional knowledge and, consequently, encouraging healthier habits that contribute to better long-term cardiovascular health [33].

This analysis is grounded in the hypothesis that the university environment, along with the students’ sociodemographic background, plays a critical role in shaping dietary habits and body composition. These are essential elements in developing effective nutritional intervention strategies and student support programs.

The practice of mindful eating, which emphasizes awareness and attention during meals, has shown positive effects on body mass and body composition in adults [34]. Further studies highlight mindful eating as an effective method to combat emotional eating and binge episodes, promoting a healthier relationship with food and better dietary choices [33]. The integration of mindfulness techniques into nutrition education programs offers a promising approach to addressing eating disorders and fostering overall well-being, emphasizing the critical role of consciousness and intention in regulating eating behaviors and maintaining a healthy weight. The symbiotic relationship between regular physical activity, healthy dietary patterns, and their impact on body weight and cardiovascular health among university students reinforces the importance of promoting healthy lifestyles [6].

This phenomenon reflects the need for personalized nutritional interventions that consider the diversity of students’ experiences and contexts to encourage healthy habits and reduce the negative effects of stress on their diets and, consequently, on their academic performance.

These programs should focus on promoting healthy habits and mitigating the negative impacts of stress and irregular physical activity on cardiovascular health and body composition in university students.

This research represents a valuable contribution to the existing body of knowledge, offering key insights for the development of policies and practices aimed at improving the health and well-being of university students.

This research thoroughly addresses the influence of diet and sociodemographic factors on the body structure and composition of university students in public higher education institutions. Through a rigorous methodological approach, the study reveals that only a small proportion (2%) of participants report a nutrient-rich diet, while 29% do not consume fats in their daily intake.

The findings indicate no significant gender differences in food and nutrient intake, which contrasts with previous research suggesting a correlation between muscle mass and total energy intake in specific student populations. However, this study highlights a notable association between fat intake and arm circumference in women, suggesting that a low-fat diet may be linked to greater muscle mass.

Furthermore, the study emphasizes the importance of sociodemographic factors such as place of birth and socioeconomic environment, particularly in men, in relation to body composition aspects like hip circumference and body weight. This underscores the influence of dietary traditions and access to healthy food, which vary considerably across regions.

The impact of academic stress on students’ eating habits is also considered, suggesting that it may lead to less healthy dietary choices with negative consequences for both physical health and academic performance. The results highlight the complexity of factors affecting the nutrition and health of university students, reinforcing the need for nutritional intervention strategies and educational programs tailored to the diverse experiences and contexts of the student population.

## 5. Conclusions

In conclusion, this study provides significant evidence on how diet and sociodemographic factors influence body composition in university students, calling for the implementation of programs that promote healthy habits and mitigate the effects of stress and irregular physical activity patterns on body composition.

## Figures and Tables

**Table 1:** Anthropometric characteristics of the study group.

	Mean	Standard Deviation	Median	Minimum	Maximum
Skinfolds (mm)	80	27.05	80	45.5	115.5
Bone diameters (cm)	102.47	3.81	102.8	96.5	107.1
Arm ratio (cm)	32.37	5.15	34.3	25.7	36.7
Waist circumference (cm)	84.17	11.7	87.3	66.5	96.0
Hip circumference (cm)	108.13	12.38	111.4	90	124
Fat mass (%)	39.93	9.01	41.0	25.8	49.4
Muscle mass (%)	23.22	3.96	23.2	18.8	29.3
Weight (kg)	72.82	14.67	75.35	53.5	91.4

**Table 2:** Results of the one-way ANOVA between diet and body composition and structure (*Pr*(>*F*) < 0.09).

Variable	df	SS	F	Pr(>F)	df	SS	F	Pr(>F)
		**WOMEN**				**MEN**		
Sum of four bone diameters	4	288	0.603	0.661	4	735	1.015	0.406
Sum of four skinfolds	4	1326	0.872	0.484	4	1467	0.741	0.567
Arm circumference	4	115.7	2.270	**0.0665** *	4	44.4	0.873	0.485
Waist circumference	4	92	0.299	0.878	4	164	0.433	0.785
Hip circumference	4	180	0.698	0.595	4	121	0.584	0.675
% Total body fat	4	168	0.794	0.532	4	69	0.270	0.896
% Total muscle mass	4	43.4	1.311	0.271	4	60.9	0.576	0.681
Body weight	4	410	0.876	0.481	4	350	0.539	0.708
Variable	df	SS	F	Pr(>F)	df	SS	F	Pr(>F)
		WOMEN				MEN		
**Dietary consumption of fruits, vegetables, and fiber**								
Sum of four bone diameters	2	65	0.274	0.761	2	294	0.807	0.451
Sum of four skinfolds	2	1529	2.058	0.133	2	472	0.477	0.623
Arm circumference	2	32.6	1.226	0.297	2	7.2	0.280	0.757
Waist circumference	2	192	1.278	0.283	2	96	0.513	0.601
Hip circumference	2	130	1.018	0.365	2	3	0.025	0.976
% Total body fat	2	64	0.600	0.550	2	72	0.579	0.563
% Total muscle mass	2	37.8	2.308	0.104	2	28.8	0.553	0.578
Body weight	2	337	1.456	0.238	2	140	0.436	0.648

Quadratic sum. * Numbers in bold are considered statistically significant when they are less than 0.05.

**Table 3:** Results of the one-way ANOVA between participants’ sociodemographic factors and body composition and structure (*Pr*(>*F*) < 0.09).

Variable	df	SS	F	Pr(>F)	df	SS	F	Pr(>F)
		**WOMEN**				**MEN**		
Sum of four bone diameters	2	412	1.777	0.174	2	109	0.295	0.745
Sum of four skinfolds	2	676	0.891	0.413	2	4111	4.663	**0.0127** *
Arm circumference	2	5.2	0.192	0.825	2	36.6	1.471	0.237
Waist circumference	2	242	1.623	0.202	2	396	2.233	0.115
Hip circumference	2	110	0.860	0.426	2	441.2	4.873	**0.0106** *
% Total body fat	2	31	0.290	0.749	2	438	3.854	**0.0261** *
% Total muscle mass	2	5.7	0.338	0.714	2	38.9	0.750	0.476
Body weight	2	68	0.289	0.750	2	812	2.695	0.0749

* Numbers in bold are considered statistically significant when they are less than 0.05.

**Table 4:** Results of the one-way ANOVA between the participants’ parental sociodemographic factors and body composition proportions (*Pr*(>*F*) < 0.09).

Variable	df	SS	F	Pr(>F)	df	SS	F	Pr(>F)
		**Women**				**Men**		
Sum of four bone diameters	2	330	1.418	0.247	2	12	0.033	0.968
Sum of four skinfolds	2	453	0.594	0.554	2	2234	2.383	0.100
Arm circumference	2	47.4	1.805	0.169	2	27.9	1.108	0.336
Waist circumference	2	33	0.216	0.806	2	200	1.090	0.342
Hip circumference	2	90	0.702	0.498	2	90	0.894	0.414
% Total body fat	2	38	0.362	0.697	2	156	1.278	0.285
% Total muscle mass	2	33.7	2.050	0.134	2	124.2	2.518	0.0882
Body weight	2	239	1.026	0.362	2	371	1.179	0.314

**Table 5:** Factor loadings of the variables onto the components.

Variables.	Comp. 1	Comp. 2	Comp. 3	Comp. 4
Fat intake score			0.460	
Fruit, vegetable, and fiber intake score			0.447	
Respondent’s place of residence		−0.460		
Father’s place of origin (irrelevant*)*		−0.501		
Mother’s place of origin (irrelevant*)*		−0.520		
Respondent’s place of birth		−0.489		
Sum of four skinfolds	−0.444			
Sum of four bone diameters				−0.454
Relaxed arm circumference	−0.417			
Waist circumference	−0.466			
Hip circumference	−0.429			
Total body fat percentage				0.489
Total muscle mass percentage				0.367
Body weight			0.401	

## Data Availability

The data used for this project can be requested from the corresponding author at tannia.carpio@espoch.edu.ec.
